# Cytological and Histopathological Spectrum of Histoplasmosis: 15 Years of Experience in French Guiana

**DOI:** 10.3389/fcimb.2020.591974

**Published:** 2020-10-29

**Authors:** Kinan Drak Alsibai, Pierre Couppié, Denis Blanchet, Antoine Adenis, Loïc Epelboin, Romain Blaizot, Dominique Louvel, Félix Djossou, Magalie Demar, Mathieu Nacher

**Affiliations:** ^1^ Department of Pathology, Centre Hospitalier de Cayenne Andrée Rosemon, Cayenne, French Guiana; ^2^ Centre of Biological Resource (CRB Amazonie), Centre Hospitalier de Cayenne Andrée Rosemon, Cayenne, French Guiana; ^3^ Department of Dermatology, Centre Hospitalier de Cayenne Andrée Rosemon, Cayenne, French Guiana; ^4^ DFR Santé, Université de Guyane, Cayenne, French Guiana; ^5^ Laboratory, Centre Hospitalier de Cayenne Andrée Rosemon, Cayenne, French Guiana; ^6^ CIC INSERM 1424, Centre Hospitalier de Cayenne Andrée Rosemon, Cayenne, French Guiana; ^7^ 1 Service des Maladies Infectieuses et Tropicales, Centre Hospitalier de Cayenne Andrée Rosemon, Cayenne, French Guiana; ^8^ Service de Médecine B, Centre Hospitalier de Cayenne Andrée Rosemon, Cayenne, French Guiana; ^9^ UMR Tropical Biome and Immunopathology, Université de Guyane, Cayenne, French Guiana

**Keywords:** histoplasmosis, pathology, HIV, French Guiana, tuberculoid granuloma, diagnosis

## Abstract

**Background:**

Disseminated histoplasmosis remains a major killer of immunocompromised patients in Latin America. Cytological and histological methods are usually present in most hospitals and may represent a precious diagnostic method. We report 15 years of experience of the department of pathology of the Centre Hospitalier de Cayenne Andrée Rosemon in French Guiana.

**Methods:**

Specimens from live patients from January 2005 to June 2020 with the presence of *H. capsulatum* on cytological and/or histological analysis were analyzed. All specimens were examined by an experienced pathologist. The analysis was descriptive.

**Results:**

Two hundred two cytological and histological samples were diagnosed with histoplasmosis between January 2005 and June 2020. The 202 samples included 153 (75.7%) histopathological formalin-fixed and paraffin-embedded tissues (biopsy or surgical specimens) and 49 (24.3%) cytological analysis from all organs. One hundred thirty-four patients (82.7%) were HIV-positive, 15 patients (9.3%) had immunosuppressant treatment, and 13 patients (8%) were immunocompetent. Seventy-eight of 202 (38.5%) were samples from the digestive tract, mostly the colon (53/78 cases, 70%) and small intestine (14/78 cases, 18%). Microorganisms were more numerous in digestive samples (notably the colon) than in other organs. Lymphocyte and histiocyte inflammation of moderate to marked intensity were observed in all positive specimens. Tuberculoid epithelioid granuloma were present in 16/78 (20,5%) specimens including 14 colon and 2 small intestine specimens. There were 11/202 cases of liver histoplasmosis, 26/202 (12,8%) cases of pulmonary histoplasmosis. Bone marrow involvement was diagnosed in 14 (2%) specimens (8 aspiration and 6 biopsies). Lymph nodes were positive in 42 specimens (31 histology and 11 cytology). Histopathological analysis of the 31 lymph nodes showed a variable histological appearance. Tuberculoid forms were most frequent (24/31, 77,4%).

**Conclusions:**

From the pathologist perspective, this is the largest series to date showing that digestive involvement was the most frequent, usually with a tuberculoid form and a greater load of *Histoplasma*. With awareness and expertise, cytology and pathology are widely available methods that can give life-saving results in a short time to help orient clinicians facing a potentially fatal infection requiring prompt treatment.

## Introduction

*H. capsulatum* is a dimorphic saprophytic fungus which exists in its mycelial form in soil at moderate temperature, ideally in a moist environment. As a soil fungus, it is well adapted to be pathogenic to humans because it does not need to interact with a mammalian host as part of its life cycle. In tissues and after inhalation of airborne conidia, *H. capsulatum* remains in the blastospore state and does not produce filaments, and is usually intracellular (Woods et al., 2001). Morphologically, *H. capsulatum* is a small spherical or ovoid yeasts measuring 2 to 6 μm (Silverman et al., 1955). It is characterized by its ability to make a dimorphic transition from must-leaven to yeast, to enter host macrophages (also called histiocytes in tissues), and to survive intracellularly. It is also able to proliferate during active infection, and to persist during clinically inapparent infection, and has the ability to reactivate (Woods et al., 2001).

Histoplasmosis was first described by Samuel Taylor Darling by examining the autopsies of two Caribbean and one Asian persons living and working in Panama who developed disseminated histoplasmosis (Darling, 1906). Since then, histoplasmosis has been attributed to *H. capsulatum*. Until recently, following different clinical and epidemiological profiles, the classification entailed *Histoplasma capsulatum* var. *capsulatum* (*H. capsulatum*), which causes classic histoplasmosis, *Histoplasma capsulatum* var. *duboisii* (*H. duboisii*) which causes African histoplasmosis, and *H. capsulatum* var. *farciminosum*, which causes lymphangitis in horses in the Old World. Insights from phylogenetic studies have now replaced this classical denomination and shown that the causal pathogens of histoplasmosis include different regionally specific cryptic species, a classification that gradually gains in detail as the sampling of isolates from different parts of the world grows (Teixeira et al., 2016; Sepúlveda et al., 2017; Damasceno et al., 2019). African histoplasmosis, is located primarily in cutaneous and subcutaneous tissue (Cockshott and Lucas, 1964). Its evolution is slow and the prognosis is benign, and the spread to the viscera remains exceptional (Zida et al., 2015).

For American Histoplasmosis, different genotypes may be associated with differences in clinical phenotype, with South American genetic lineages being more dermotropic, and leading to acute pulmonary diseases whereas North American strains would be linked to more chronic pulmonary disease. These differences however, may be confounded by differences in access to care and further studies are still needed to disentangle what is attributable to differences in Histoplasma genetics and patient health care trajectories (Couppie et al., 2006; Morote et al., 2020; Rodrigues et al., 2020).

The severity of disease after inhalation of *H. capsulatum* varies, with the intensity of exposure and the host’s immunity. This may lead to asymptomatic infections or mild pulmonary disease for low-intensity exposures in immunocompetent individuals, whereas heavy exposures may lead to severe pulmonary infections. Among patients with underlying lung disease, a chronic lung infection may develop with gradual loss of pulmonary function, and if untreated, frequent death. Among immunocompromised patients the infection progressively disseminates to other organs leading to non-specific syndromes. Various organs such as the lungs, gastrointestinal tract, liver or lymph nodes may thus be involved in the same patient. Immunocompromised patients infected with *Histoplasma* are 10 to 15 times more likely to develop a disseminated form of the disease, which is invariably fatal if left untreated. Hence, HIV-infected patients with disseminated histoplasmosis usually have CD4 counts under 200 per mm^3^ and mostly under 50 per mm^3^. In the past four decades, the acquired immune deficiency syndrome (AIDS) pandemic and the rise in the number of pharmacologically immunosuppressed patients have led to an increase of the population of patients susceptible to progressive disseminated histoplasmosis, and a high number of resulting fatalities in a context where diagnosis is difficult and antifungal treatment is often absent or late (Pan American Health Organization, 2020).

Histoplasmosis has thus become a major opportunistic infection in patients with advanced HIV infection in endemic areas, where it has often been ranked as the most common AIDS-defining infection and cause of death for patients with CD4 cell counts <200 cells/mm^3^ (Nacher et al., 2011; Nacher et al., 2020).

French Guiana combines a favorable environment for the growth of fungi with a high prevalence of HIV infection (Nacher et al., 2018a). Epidemiological statistics suggest that the incidence of disseminated histoplasmosis in HIV patients is as high as 1.5 per 100 persons-years overall, and greater than 10 per 100 persons-years in patients with CD4 counts under 50/mm^3^ (Nacher et al., 2011; Nacher et al., 2014). In South America, histoplasmosis is a common diagnosis, frequently considered by physicians confronted with HIV patients with symptoms of an infectious disease, and pulmonary or digestive symptoms (Couppié et al., 2019).

Before the advent of the AIDS epidemic, histoplasmosis data were obtained from animal samples (Emmons and Ashburn, 1948) or by including autopsy specimens sometimes several decades after the initial diagnosis (Queiroz and Siqueira, 1975).

Various techniques have been used to detect Histoplasma infection. The reference methods are cytological and histological examination and fungal culture with a sensitivity of 65 and 80% respectively in immunocompromised patients (Wheat, 2003; Wheat, 2006; Nacher et al., 2018b). Other common procedures include DNA detection by polymerase chain reaction (PCR), and serological detection in plasma. However, the sensitivity of histoplasmosis detection methods reported in the literature are highly variable, and may differ geographically and according to the experience and the laboratory method used in each center (Wheat, 2003; Tobon et al., 2005; Huber et al., 2008; Mata-Essayag et al., 2008). In addition, the relevance of different tests may vary according to the organs retrieved or the immune status of the patient (Tobon et al., 2005; Weydert et al., 2007). Interestingly, the detection of the *H. capsulatum* antigen in serum or urine has shown a higher sensitivity, estimated at 90% on American strains. However, access to this method is limited and is only available in certain North American laboratories. Because of the diagnostic gap in most countries, most studies highlighting pathological findings of histoplasmosis are usually presented in the form of case reports. Some cohorts with larger numbers of patients have been assembled in the United States in successive epidemics of the east-central regions concerning mainly immunocompetent patients (Cano and Hajjeh, 2001). At the same time, the incidence has been steadily increasing in parts of South America, where access to medical care may be more difficult. Whereas fungal culture, antigen detection, or PCR are often not available, cytological and histological methods are usually present in most hospitals in endemic countries and may represent a precious diagnostic alternative. In this review, we report the experience of the department of pathology of the Centre Hospitalier de Cayenne Andrée Rosemon in French Guiana in terms of microscopic diagnosis of *H. capsulatum* histoplasmosis for more than 15 years (2005 and 2020) carried out on cytological and histological samples.

## Methods

We analyzed specimens from live patients diagnosed with histoplasmosis from January 2005 to June 2020 at the Centre Hospitalier de Cayenne Andrée Rosemon in the department of pathology (Cayenne, French Guiana). The inclusion criteria were the confirmed presence of *H. capsulatum* histoplasmosis on cytological and/or histological analysis. All specimens were examined by an experienced pathologist to confirm the initial diagnosis by looking for *H. capsulatum* organisms, such as the typical round intracellular yeast 2–6 μm with buds. All solid tissue samples were fixed in 10% buffered formalin, embedded in paraffin, sectioned at 4 µm and stained with routine Hematoxylin-Eosin-Safran (HES) stain as well as with Silver Methenamine Gomori-Grocott (Gomori-Grocott) and/or Periodic-Acid-Schiff (PAS) stain. The yeasts of *H. capsulatum* are usually colored red-violet and surrounded by a light halo by PAS stain, and colored brown-black by the Gomori-Grocott stain.

The histopathological lesions correspond to the host reactions against *H. capsulatum* and its immune status, and are classified into 4 categories: (i) the tuberculoid form, (ii) the anergic form, (iii) the mixed form, and (iv) the sequelae form.

The tuberculoid form usually corresponds to a low inoculation and effective tissue response of the host. The tissue shows an inflammatory infiltrate rich in activated macrophages and lymphocytes mostly of T-helper CD4+ phenotype progressively recruited *in vivo*. Granulomas with epithelioid cells with/without giant cells can also be observed. The evolution of this form, by analogy with tuberculosis, is caseous necrosis. Here, the histoplasmas are usually few in number and are located in the cytoplasm of histiocytes (intracellular).

The anergic form is observed in HIV patients and shows little or no tissue response. Local macrophages remain inactive. The typical appearance is that of an abundance of intracellular and extracellular yeast.

The mixed form represents an intermediate form between the tuberculoid form and the anergic form. Finally, in the sequelae form, the scarring fibrosis are predominates and the inflammation is mild. In this form, rare yeasts can be found that can correspond to a relapse case or eventual reactivation.

### Ethical and Regulatory Aspects

HIV-infected patients were enrolled in the French Hospital Database for HIV (FHDH). The database includes most patients followed in French Guiana and nearly all AIDS cases. Patients in the FHDH gave informed consent for using their anonymized data and for publishing anonymized results. This data collection was approved by the Commission Nationale Informatique et Libertés (CNIL) since 1992 and this cohort has led to multiple international publications. For HIV-negative patients, posters and leaflets in a range of language were posted in laboratories and wards to inform patients that their anonymized results may be used in ancillary studies and that they have a right to refuse without any impact on access and quality of care.

## Results

Two hundred two cytological and histological samples (from 162 patients) of *H. capsulatum* histoplasmosis were diagnosed in our department between January 2005 and June 2020. The 202 samples included 153 (75.7%) histopathological formalin-fixed and paraffin-embedded tissues (biopsy or surgical specimens) and 49 (24.3%) cytological analysis from all organs. One hundred thirty-four patients (82.7%) were HIV-positive, 15 patients (9.3%) had immunosuppressant treatment (long-term steroids, anti-TNF and chemotherapy), and 13 patients (8%) were immunocompetent with no known immunodeficiency status at the time of diagnosis. In HIV cases, the CD4+ cell count at the time of cytological or histopathological diagnosis ranged from 0 to 668 mm^3^. **Table 1** summarizes histopathological data of 153 *H. capsulatum* positive tissues and **Table 2** summarizes the 49 cytological techniques used for detection of *H. capsulatum*.

**Table 1:** This table summarizes histopathological data of 153 *H. capsulatum* positive tissues.

Organ/histological type	N°	Tuberculoid type	Anergic type	Intermediate type	Sequelae type
**Gastro-intestinal tract**	78	16/78 (20,5%)	17/78 (21.8%)	40/78 (51.2%)	5/78 (6.5%)
**Liver**	11	9/11 (82%)	2/11 (18%)	0/11 (0%)	0/11 (0%)
**Lung**	2	2/2 (100%)	0/2 (0%)	0/2 (0%)	0/2 (0%)
**Bone marrow**	6	4/6 (66,6%)	1/6 (16.7%)	1/6 (16.7%)	0/6 (0%)
**Lymph node**	31	24/31 (77,4)	4/31 (12.9%)	3/31 (9.7%)	0/31 (0%)
**Skin**	16	9/16 (56,3)	2/16 (12.5%)	4/16 (25%)	1/16 (6.2%)
**ENT**	7	4/7 (57,1%)	1/7 (14.3%)	2/7 (28.6%)	0/7 (0%)
**Joints**	2	1/2 (50%)	0/2 (0%)	1/2 (50%)	0/2 (0%)
**TOTAL**	**153**	**69/153**	**27/153**	**51/135**	**6/153**

The histopathological lesions correspond to the host reactions against H. capsulatum and its immune status, and are classified into 4 categories: (a) the tuberculoid type, (b) the anergic type, (c) the intermediate type, and (d) the sequelae type.

**Table 2:** This table summarizes the 49 cytological techniques used for detection of *H. capsulatum* in our series.

Organ	N°	Cytological technique
**Lung**	24 (49%)	Bronchoalveolar lavage (BAL)
**Bone marrow**	8 (16.3)	Bone marrow aspiration
**Lymph node**	11 (22.5%)	Fine needle aspiration (FNA) of lymph node
**CNS**	1 (2%)	Cerebro-spinal fluid/cytology
**Peritoneum**	3 (6.2%)	Peritoneal fluid/cytology
**Prostate**	1 (2%)	Fine needle aspiration (FNA) of inflammatory prostatic lesion
**Blood**	1 (2%)	Peripheral blood smear
**TOTAL**	**49**	

### Pulmonary Histoplasmosis

We diagnosed pulmonary histoplasmosis in 26/202 (12,8%) specimens consisting of 24 BAL and 2 bronchial biopsies. The two bronchial biopsies involved two HIV patients with CD4 at 124 and 30 mm^3^. Both samples revealed a tuberculoid form associated with intra-cellular *H. capsulatum* (PAS+ and/or Gomori-Grocott +).

The 24 BAL fluid concerned 23 immunocompromised patients and one immunocompetent patient. Among the 23 immunocompromised patients with a BAL, there were 22 HIV patients and one post-renal transplant patient treated with immunosuppressant drugs who had a BAL because he was suffering from fever and diffuse pulmonary micronodules.

**Figure 1:**
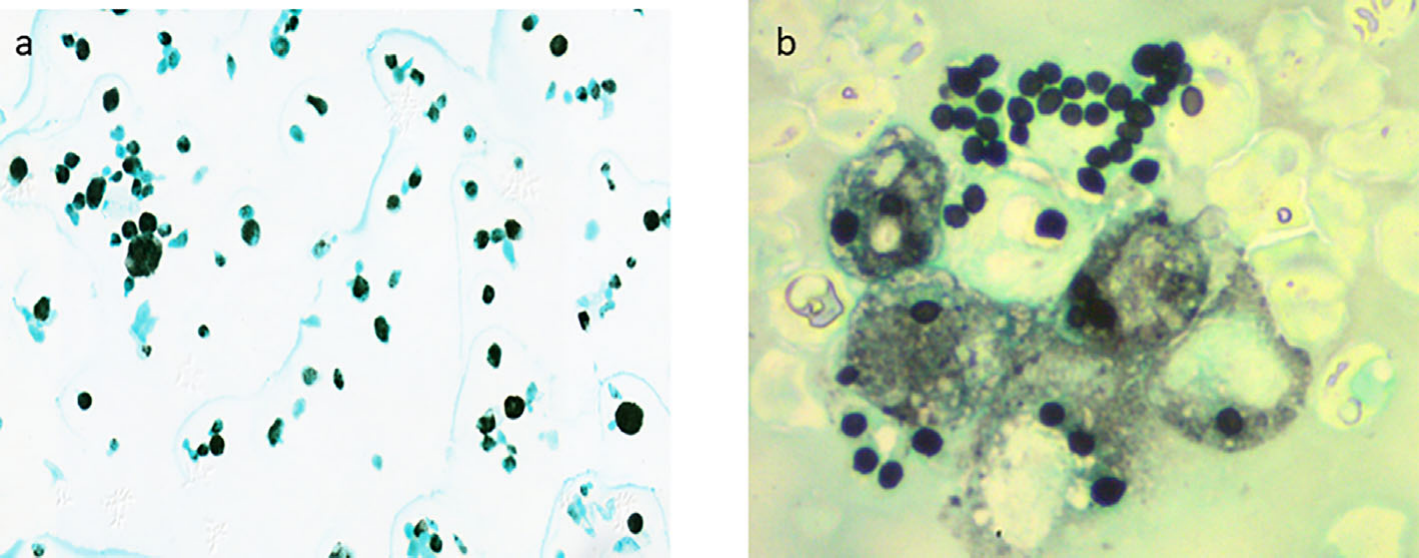
*H. capsulatum* is a small spherical or ovoi yeasts measuring 2 to 6 μm characterized by its ability to make a dimorphic transition to enter host macrophages and to survive intracellularly and proliferate during active infection. **(A)** Pulmonary histoplasmosis: BAL cytology shows macrophages with numerous intracellular *H. capsulatum* (Gomori-Grocott stain x400). **(B)** Disseminated histoplasmosis: Extracellular *H. capsulatum* from blood smear (Gomori-Grocott stain x1500).

The cellular formula of all the 24 pathologic BAL consisted essentially of macrophages with a percentage ranging from 55 to 85% of cellularity (normal >80%). Neutrophils were in second place with 2 to 30% of cellularity (normal <5%), followed by lymphocytes with 8 to 18% of cellularity (normal <10%). The presence of plasmocytes and eosinophils was occasional. **Figure 1** shows a case of pulmonary histoplasmosis diagnosed on BAL.

We routinely performed Ziehl-Neelsen staining and immunochemistry study with the anti-*Pneumocystis jiroveci* antibody on BAL and bronchial biopsies of HIV patients to look for a co-infection with tuberculosis and pneumocystosis respectively. Histoplasmosis was associated with the presence of *Mycobacterium tuberculosis* on the same cytology specimen in two cases, *Pneumocystis jiroveci* in one case and candidiasis in two cases.

### Bone Marrow Histoplasmosis

Bone marrow involvement by histoplasmosis was diagnosed in 14 (2%) specimens (8 aspiration and 6 biopsies). It was frequently associated with cytopenia (anemia, neutropenia, and thrombocytopenia) and sometimes with pancytopenia. The tuberculoid form without necrosis was present in 4 of 6 bone marrow biopsies. The two remaining biopsies revealed an anergic form in one case, and intermediate form in the second. **Figure 1** shows a case of disseminated histoplasmosis diagnosed from a blood smear.

### Lymph Node Histoplasmosis

**Figure 2:**
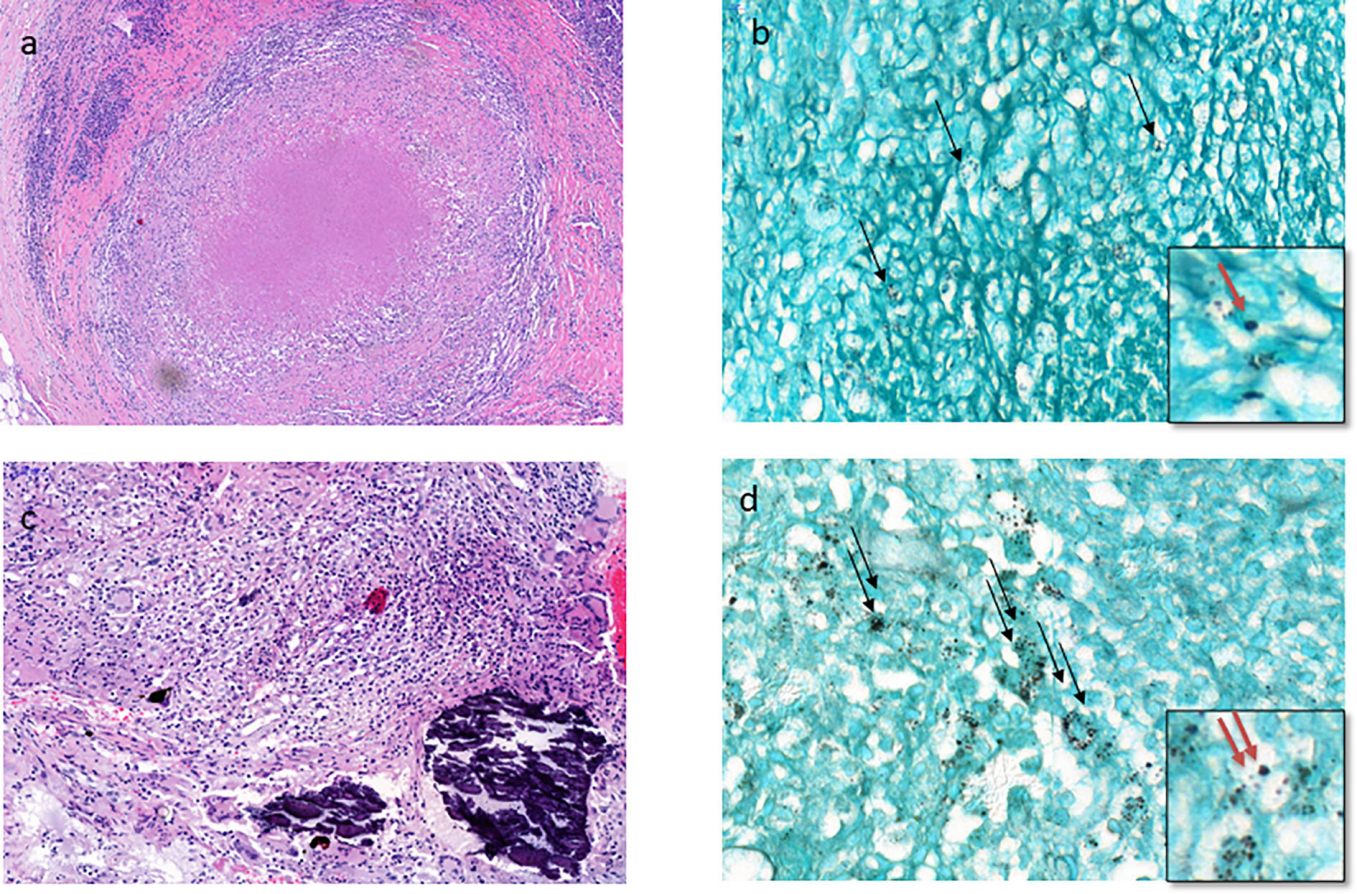
The tuberculoid type of histoplasmosis. **(A)** Lymph node tuberculoid granuloma perfectly mimicking tuberculosis with epithelioid cells, multinucleated giant cells, and caseous necrosis (HES stain x200). **(B)** Few intracellular *H. capsulatum* (black arrow) in the peripheral layers of tuberculoid granuloma of the lymph node (Gomori-Grocott stain x 400) with focus spot (red arrow x1000). **(C)** Branchial biopsy shows less typical granuloma with macrophages, some epithelioid cells, and few multinucleated giant cells and calcifications without necrosis (HES stain x400). **(D)** Moderate number of intracellular *H. capsulatum* (two black arrows) from the same branchial biopsy (Gomori-Grocott stain x 600) with focus spot (two red arrows x1000).

Microscopic analysis confirmed lymph node involvement by *H. capsulatum* in 42 specimens (31 histology and 11 cytology). The 11 histological samples consisted of 2 biopsies and 9 surgical specimens of lymph nodes. Histopathological analysis of the 31 lymph nodes showed a variable histological appearance. The tuberculoid form was the most frequent (24/31, 77,4%). Interestingly, a histological variant perfectly mimicking tuberculosis with epithelioid granulomas, multinucleated giant cells, and caseous necrosis was found in 10 of the 24 tuberculoid forms, followed by a less typical granulomatous variant where giant cells and necrosis were absent in 8 cases, and then a non-granulomatous histiocytic inflammation form in 6 cases (**Figure 2**). The remaining 7 lymph nodes showed non-specific follicular or sinusal histiocytic hyperplastic lymphadenitis.

In lymph node involvement (cytological and histological specimens), *H. capsulatum* were found with Gomori-Grocott and PAS+ special staining, which were routinely performed in HIV patients.

The Ziehl-Neelsen stain allowed the diagnosis of a histoplasmosis-tuberculosis coinfection in 3 cases. One case of lymph node histoplasmosis was associated with T-cell lymphoma in a patient with HTLV-1 infection.

### Digestive Histoplasmosis

In our experience the digestive tract was the most affected organ by histoplasmosis. Seventy-eight of 202 positives samples (38.5%) belonged to the digestive tract (73 biopsies and 5 surgical specimens). Multiple ulcers of the digestive mucosa were the most common endoscopic findings (56/78 cases, 71.7%). Three of the five surgical specimens involved a parietal perforation and two cases involved a pseudotumoral fibro-inflammatory intestinal occlusion.

The colon was the organ most affected by histoplasmosis (53/78 cases, 70%) followed by the small intestine (14/78 cases, 18%). The other sites were less frequently involved: esophagus (3/78 cases, 3.8%), stomach (4/78 cases, 5.2%), rectum (2/78 cases, 2.5%), and anal region (2/78 cases, 2.5%). Concomitant colic and intestinal involvement were observed in 6 cases, and both colic and esophageal involvement in only 1 case.

**Figure 3:**
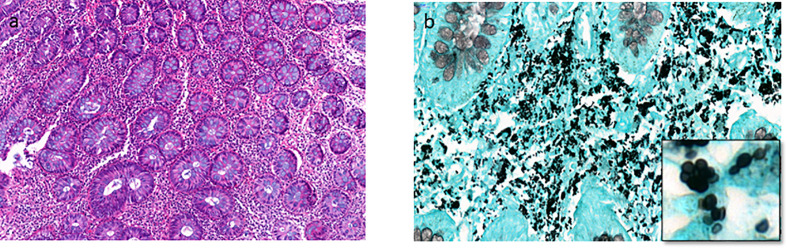
The anergic type of histoplasmosis. **(A)** Colon biopsy shows interstitial moderate and polymorph inflammatory infiltrate including lymphocytes, plasmocytes, and some macrophages (HES stain x200). **(B)** Numerous intracellular and extracellular *H. capsulatum* from the same biopsy (Gomori-Grocott stain x600, with focus spot x1500).

The numbers of microorganisms were higher in digestive histoplasmosis than in other organs, with a maximum for colonic biopsies (number of *H. capsulatum* per light microscope field at magnification ×400). In addition, in these samples, *histoplasma* were observed in the cytoplasm of histiocytes or free in the stroma. Lymphocyte and histiocyte inflammation of moderate to marked intensity were observed in all positive specimens. Nevertheless, the tuberculoid epithelioid granulomatous form was present in 16/78 (20,5%) specimens including 14 colon and 2 small intestine specimens. The presence of multinucleated giant cells was observed in only 3 of the 16 cases. *H. capsulatum* yeasts were present mainly in the peripheral layers near the granuloma. No granulomas were found in the upper gastrointestinal tract. In addition, mixed inflammatory infiltrate contained plasma cells, neutrophils, and eosinophils were observed. **Figure 3** shows an anergic form of histoplasmosis in the colon.

### Liver Histoplasmosis

Eleven cases of liver histoplasmosis were diagnosed in our department in the last 15 years. All eleven patients were HIV-positive and had fever, cholestasis and an altered general condition. On histological examination, the hepatic tissue was the site of moderate to marked lymphohistiocytic inflammatory infiltrates and/or epithelioid granulomas without necrosis (tuberculoid form) in 9 out of 11 cases. The two remaining cases corresponded to a rather anergic form. Sinusoidal hyperplasia of Kupffer cells was also observed in 5 cases. *H. capsulatum* were intra-cellular and occasionally observed in the sinusoid lumen in both cases of the anergic form.

### Mucocutaneous Histoplasmosis

**Figure 4:**
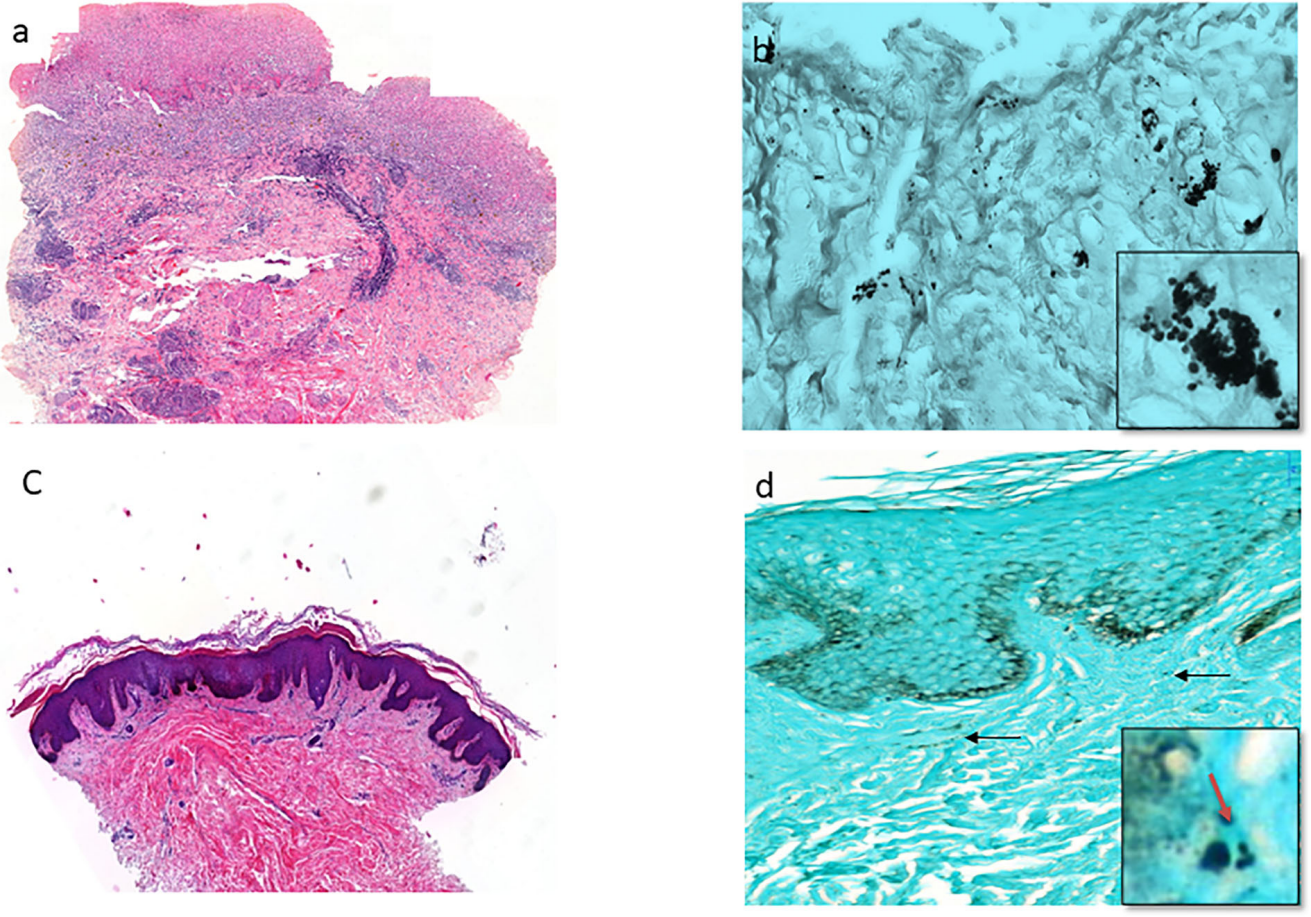
The cutaneous histoplasmosis (intermediate and sequalae types). **(A)** Ulcerative skin biopsy shows marked polymorph dermatitis including neutrophils, lymphocytes, plasmocytes, eosinophils, and few macrophages (HES stain x200). **(B)** Presence of moderate number of intracellular and extracellular *H. capsulatum* in the dermis from the same biopsy (Gomori-Grocott stain x600, with focus spot x1000). **(C)** Skin biopsy shows a particularly fibrous dermis without significant inflammatory infiltration in an HIV patient (HES stain x100). Gomori-Grocott staining shows very rare *H. capsulatum* (black arrow) in the dermis (Gomori-Grocott stain x600, with focus spot and red arrow x1000).

Histological analysis revealed the presence of *H. capsulatum* microorganisms in 16 mucocutaneous biopsies (12 skin biopsies, 3 oral mucosa biopsies, and 1 genital mucosa biopsy). Interestingly, the tuberculoid form associating epithelioid granulomas and multinucleated giant cells was the most frequent form (9/15, 60%). Necrosis was consistently absent. The intermediate form was observed in 6 cases (40%). **Figure 4** shows intermediate and sequelae cases of cutaneous histoplasmosis.

Diverse localizations of histoplasmosis were diagnosed by the Pathology Department in the last 15 years including cerebrospinal fluid which correspond to central nervous system involvement (1 case), peritoneal fluid (3 cases), cytology from a prostatic lesion (1 case), and peripheral blood smear in a patient with disseminated histoplasmosis. Seven biopsy specimens of ENT histoplasmosis were observed in our series (3 larynx, 1 cavum, 1 nasal fossa, 1 tonsil, and 1 tongue). We also diagnosed two synovial histoplasmoses, one of them was the subject of a recent case report (Gaume et al., 2017).

## Discussion

In the pathology department of the Centre Hospitalier de Cayenne Andrée Rosemon in French Guiana, consecutive patients diagnosed with histoplasmosis were almost exclusively immunocompromised, mainly from advanced HIV-infection. The appearance of histoplasmosis in patients suffering from cancer or treated with immunosuppressive drugs also emphasizes that this infection should be systematically suspected in endemic areas, a suspicion that should lead to specific explorations to identify it.

In this review of over 15 years of activity in the main hospital in French Guiana, we have reported the cytopathological and histopathological aspects of histoplasmosis diagnosed by light microscopy. To our knowledge, this is the largest series of pathological specimens diagnosed with histoplasmosis. The frequency of different types of samples may have reflected local organizational specificities. Hence, first clinicians must be proactive to obtain tissue samples for fungal culture or pathology. When the sample is taken it should be divided and immersed in formalin for pathology whereas it should not for fungal culture. These aspects are not trivial, they require awareness and a specific organization that allows patients and physicians to benefit from at least two complementary diagnostic methods. In French Guiana early on, physicians, and notably gastroenterologists have always been prompt to perform endoscopies to explore diarrheal diseases of HIV thereby identifying a significant number of cases of histoplasmosis, which presumably explains the large number of cases in the digestive tract.

We described the pathological findings in an immunodeficient population whose immune condition may have specificities.

We revealed that *Histoplasma* can be found in and beneath the gastrointestinal mucosa in patients who present multiple endoscopic ulcers. The ulcers were associated with a large number of *H. capsulatum* yeasts and fever. In ulcers, yeast was often present outside the macrophages or even linked in small chains, a likely sign of intense fungal replication and/or tissue necrosis.

The tuberculoid form was the most common histological form found across all organs. This granulomatous form is found in both immunocompetent and immunocompromised patients. Nevertheless, the typical tuberculoid appearance associating both well-formed epithelioid granulomas and multinucleated giant cells with or without caseous necrosis was not so frequent, a finding that has already been highlighted by previous studies performed only on digestive and hepatic histoplasmosis (Lamps et al., 2000). Granulomas are strongly associated with a high fungal load, which easily leads to diagnosis. However, low fungal loads with <1 *H. capsulatum* yeast per microscopic field ×400, without any real inflammatory infiltrate, are not uncommon and should be diagnosed using the Gomori-Grocott stain, an oil lens with a higher magnification (×1,000) to avoid confusion with other fungal yeasts.

In this series, we reported two cases of histoplasmosis diagnosed following colon and small intestine occlusion by chronic fibro-inflammatory masses of pseudotumoral appearance. Given the diversity of the fungal load and inflammation on the slides, pathologists must be experienced in the identification of this yeast. Overall, the rarity of fibrosis and predominance of tuberculoid forms suggests that the evolution of infections may usually be subacute (1 to 3 months) because fibrotic lesions are usually associated with chronic infections (6 months). The rarity of fibrosis is presumably also a consequence of immune suppression which reduces inflammation. This is consistent with the observation of seasonality which suggested that a significant proportion of histoplasmosis cases were *de novo* infections (Hanf et al., 2010).

The diagnosis of histoplasmosis is not always simple. Most data have been provided from epidemics in predominantly immunocompetent North American populations with lung involvement. Patients in South America and in our series in French Guiana were mostly immunocompromised. While culture provides the strongest evidence of infection, it can be slow, despite recent improvements in culture media, and it requires BSL 3 laboratories. In addition, the antibodies currently in use are based on *H. capsulatum* strains from the United States, which may not be accurate enough for Amazonian strains. Studies have shown a remarkable polymorphism of *H. capsulatum* strains in Brazil, with genetic differences with the American strains leading to possible differences in clinical and laboratory results (Karimi et al., 2002; Zancopé-Oliveira et al., 2005).

A US study of 16 immunocompromised patients suggested that histology was the most useful diagnostic procedure (Kaufman, 1992). Since most of our patients present with severe systemic symptoms that require immediate care, a combination of several laboratory techniques such as direct MGG-stained examination, culture on Sabouraud dextrose agar, examination of pathological specimens (cytology and histology) stained by PAS or Gomori-Grocott, PCR, *Histoplasma* antigen (galactomannan antigen), or serology seems necessary. Information on the efficacy of histopathological examinations in the diagnosis of histoplasmosis is scarce. Figures found in the literature refer mainly to results obtained mainly from lung samples during the Indiana epidemics and acknowledge a sensitivity of about 60% in immunocompromised patients (Wheat, 2006). On the contrary, the results obtained in the endemic population of South America show a remarkable initial sensitivity in 158 patients including 27 immunocompromised patients (Mata-Essayag et al., 2008). This result highlights the need for pathologists experienced in the diagnosis of infectious agents.

Previous studies based on autopsy results confirm that gastrointestinal involvement was common in disseminated histoplasmosis (Silverman et al., 1955; Queiroz and Siqueira, 1975; Goodwin et al., 1980). However, it caused symptoms in only a few patients (3–12%) in older studies involving mainly immunocompetent patients (Suh et al., 2001), which probably explains why gastrointestinal specimens are rarely considered for the diagnosis of histoplasmosis in daily practice in non-endemic areas.

The gastrointestinal tract was commonly involved in the histoplasma infection. In case of suspicion of histoplasmosis in patients reporting diarrhea or other digestive symptoms, upper tract endoscopy and ileocolonoscopy were often performed in our institute. While upper gastrointestinal specimens taken at the same time of diagnosis often yielded negative results, the ileum and colon were a common site of infection. Histopathology provided valuable information and proved to be very sensitive, probably due to the high number of yeasts found there. Histological examination for digestive histoplasmosis can provide a rapid diagnosis and avoid false negativity due to possible contaminating fungal organisms such as *Candida* yeast, which grow faster than histoplasmosis in the culture broth.

Similarly, cytological and histopathological examinations showed a high sensitivity in pulmonary histoplasmosis. In our experience, cytological examination of BAL fluid allows the diagnosis of pulmonary histoplasmosis and reveals *H. capsulatum* microorganisms within macrophages by PAS and/or Gomori-Grocott staining.

In our series the tuberculoid form was the most common histopathologic findings in liver histoplasmosis biopsies (9/11 specimens, 81,8%). These data are interesting because, unlike most previously published cases, this series involved hepatic tissue samples involved living hospitalized patients and not autopsy specimens.

Bone marrow aspiration was very effective in diagnosing *H. capsulatum*, and was easily able to detect the Gomori-Grocott positive yeast.

Finally, when looking for *H. capsulatum*, the search for other pathogens especially in immunocompromised patients should not be neglected. In our series histoplasmosis was associated with other infectious diseases including toxoplasmosis, *cytomegalovirus*, *Epstein-Barr virus*, *Pneumocystis*, genital herpes, leishmaniasis, syphilis, Chagas disease, local or systemic candidiasis, hookworm disease, dengue fever, leprosy, and cryptococcosis. The discovery of systemic bacterial infection like *Salmonella*, *Pseudomonas*, and *Klebsiella* was also common during hospitalization.

Overall, our results show the good performance of pathological analyses in a population which had a predominance of immunocompromised patients, by the high number of positive cytological and histological samples, and the experience acquired by the pathology team in the daily search for *Histoplasma* microorganisms. Cytological and/or histopathological analysis with an average of 3 days between sample collection and results is a relatively rapid and reliable diagnostic tool for histoplasmosis. In combination with other laboratory analyses the pathological analysis using special routine stains (PAS and Gomori-Grocott) is particularly useful and accurate in gastrointestinal histoplasmosis of the lower digestive tract and in the lungs, but also in other locations of histoplasmosis such as liver, bone marrow, lymph nodes, and skin. Given the diversity of fungal load and the cytological and histological findings on the analyzed specimens, the pathologist must have sufficient experience to identify *H. capsulatum* and think systematically about histoplasmosis as a differential diagnosis in HIV patients. Clinicians must also be aware of the possibility of histoplasmosis and should strive to obtain tissue samples adequately processed so that both fungal culture and cyto-pathology can be performed. Hence, guided by the clinical presentation, digestive endoscopies, bone marrow and lymph node aspiration, and biopsies were contributive standard explorations. Although great hopes have emerged for the scale-up of new rapid diagnostic methods clinicians should make full use of classical methods that are available and that can help save lives.

## Data Availability Statement

The original contributions presented in the study are included in the article/supplementary material. Further inquiries can be directed to the corresponding author.

## Author Contributions

KD: study design, analysis, first draft. MN: editing and final draft. PC, DB, LE, FD, AA, RB, DL, MD: data collection and validation of analysis and manuscript. All authors contributed to the article and approved the submitted version.

## Conflict of Interest

The authors declare that the research was conducted in the absence of any commercial or financial relationships that could be construed as a potential conflict of interest.
